# Truncated *SPAG9* as a novel candidate gene for a new syndrome: Coarse facial features, albinism, cataract and developmental delay (CACD syndrome)

**DOI:** 10.1590/1678-4685-GMB-2024-0094

**Published:** 2025-01-20

**Authors:** Majid Alfadhel, Bashayr S. Alhubayshi, Muhammad Umair, Ahmed Alfaidi, Deemah Alwadaani, Essra Aloyouni, Safdar Abbas, Abdulkareem Al Abdulrahman, Mohammed Aldrees, Abeer Al Tuwaijri, Ruaa S. Alharithy, Abdulaziz Alajlan, Abdulrahman Alswaid, Saad Almohrij, Sultan Al-Khenaizan

**Affiliations:** 1 King Saud Bin Abdulaziz University for Health Sciences (KSAU-HS), Ministry of National Guard Health Affairs (MNG-HA), King Abdulaziz Medical City (KAMC), King Abdullah International Medical Research Center (KAIMRC), Medical Genomics Research Department, Riyadh, Saudi Arabia.; 2 King Saud Bin Abdulaziz University for Health Sciences, Ministry of National Guard Health Affairs (MNG-HA), King Abdulaziz Medical City (KAMC), Genetics and Precision Medicine Department (GPM), King Abdullah Specialized Children’s Hospital, Riyadh, Saudi Arabia.; 3 King Saud Bin Abdulaziz University for Health Sciences (KSAU-HS), College of Medicine, Riyadh, Saudi Arabia.; 4 Security Forces Hospital, Department of Dermatology, Riyadh, Saudi Arabia.; 5 Dartmouth College, Department of Biological Science, Hanover, NH, United States.; 6 Ministry of National Guard Health Affairs (MNG-HA), King Abdulaziz Medical City (KAMC), Department of Pathology and Laboratory Medicine, Riyadh, Saudi Arabia.; 7 Ministry of National Guard Health Affairs (MNG-HA), King Abdulaziz Medical City (KAMC), Department of Surgery, Riyadh, Saudi Arabia.; 8 Ministry of National Guard Health Affairs (MNG-HA), King Abdulaziz Medical City (KAMC), Department of Dermatology, Riyadh, Saudi Arabia.

**Keywords:** SPAG9, oculocutaneous albinism, intellectual disability, cataract, frameshift variant

## Abstract

Sperm-associated antigen 9 (SPAG9) is a member of cancer-testis antigen, having characteristics of a scaffold protein, which is involved in the c-Jun N-terminal kinase JNK signaling pathway, suggesting its key involvement in different physiological processes, such as survival, apoptosis, tumorigenesis, and cell proliferation. We identified two families (A and B) having multisystem features like coarse facial features, albinism, cataracts, skeletal abnormalities, and developmental delay. Whole genome sequencing (WGS) in families A and B revealed a homozygous frameshift variant (c.903del; p.Phe301Leufs*2) in the *SPAG9* gene. Sanger sequencing of both families revealed perfect segregation of the identified variant in all family members. 3D protein modeling revealed substantial changes in the protein’s secondary structure. Furthermore, RT-qPCR revealed a substantial reduction of *SPAG9* gene expression at the mRNA level in the affected individuals of both families, thus supporting the pathogenic nature of the identified variant. For the first time in the literature, biallelic *SPAG9* gene variation was linked to multisystem-exhibiting features like coarse facial features, albinism, cataracts, skeletal abnormalities, and developmental delay. Thus, this data supports the notion that *SPAG9* plays an important role in a multisystemic disorder in humans.

## Introduction

Sperm-associated antigen 9 (*SPAG9*) was cloned in 1998 and called a protein highly expressed in testis (PHET) (Shankar et al., 1998). SPAG9 has been implicated in signal transduction in interacting with the JNK (c-Jun N-terminal kinase) signaling pathway, which is involved in cell survival, apoptosis, stress responses and plays a vital role in maintenance of cell shape, motility, and intracellular transport. *SPAG9* is known to be expressed in various tissues, including the testes, which is consistent with its original identification as a sperm-associated antigen. It’s worth noting that *SPAG9’s* expression might vary in different cell types and tissues (Jagadish et al., 2018).

SPAG9 is a JNK-associated leucine zipper protein (JLP) and with JNK/stress-activated protein kinase-associated protein 1 (JSAP1 or MAPK8IP3), which are structurally related scaffolding proteins highly expressed in the brain. JIP4 is a scaffold protein that in humans is encoded by the *SPAG9* gene. SPAG9 plays functionally redundant and essential roles in mouse cerebellar Purkinje cell (PC) survival (Sato et al., 2015a). Mice containing PCs with deletions in both JSAP1 and JLP exhibited PC axonal dystrophy, followed by gradual, progressive neuronal loss. These findings suggest that JSAP1 (MAPK8IP3) and JLP play critical roles in kinesin-1-dependent axonal transport, which prevents brain neuronal degeneration (Sato *et al*., 2015a, 2015b). With structural homology of JNK, *SPAG9* is involved in MAPK signaling pathway to regulate cellular activities (Pan et al., 2018).

Oculocutaneous albinism (OCA) is a heterogeneous group disorder that results in either reduction or complete loss of melanin formation of melanin in melanocytes, which results in mild to severe hypopigmentation of the hair, skin, and eyes. OCA is classified into two subtypes, non-syndromic OCA and syndromic OCA. Non-syndromic OCA is caused by disease causing variants in genes associated with melanocyte differentiation, melanosomal proteins, and melanin synthesis, which cause only hypopigmentation and visual-associated symptoms (Fernández et al., 2021). Currently, there are eight types of non-syndromic OCA that have been reported: OCA1 to OCA8 and the most common forms are TYR-related types and include OCA type 1A (MIM 203100) and OCA type 1B (MIM 606952) (Manoli et al., 2010; Grønskov et al., 2013; Kausar et al., 2013; Wei et al., 2013; Morice-Picard et al., 2014; Garrido et al., 2021). Furthermore, syndromic OCA includes systemic phenotypic manifestations such as intellectual disability, global developmental delay, and cataract. Syndromic OCA is caused by mutations in cargo trafficking proteins, which contribute to the formation of lysosome-related organelles (LROs). LROs are cell type-specific organelles, such as melanosomes in melanocytes, which cause the classic OCA phenotype of skin, eye, and hair hypopigmentation. Hermansky-Pudlak syndrome (HPS) and Chediak-Higashi syndrome (CHS) are the two most common types of syndromic OCA (Garrido *et al*., 2021).

Only cutaneous and ocular signs and symptoms in OCA patients have been studied; its association with brain development and other associated phenotypes has not been studied well.

As we know that disruption of the tyrosine pathways involved in the OCA are essential for the retinal and visual network development, however indirectly, that are also linked to progressive neurodevelopment in the patients (Neveu et al., 2022).

In the present study, we aim to describe a novel syndrome for the first time in the literature, and we link an *SPAG9* gene defect as a possible cause of this syndrome (CACD syndrome). We performed genetic, clinical and molecular characterization of two families to find the associated genes and further performed functional study (RT-qPCR) and 3D protein modeling to examine the pathogenicity of the identified variant.

## Subjects and Methods

### Ethics approval and consent to participate

The study was approved by the Institutional Review Board (IRB) of King Abdullah International Medical Research Centre (KAIMRC), Riyadh [Grant # RC19/138/R; IRBC/1580/20]. The parents of the patient gave written informed consent for publications of data and images in accordance with the Declaration of Helsinki. Written informed consent for the publication of related data was obtained from the parents of the enrolled patients. 

### Patient recruitment and DNA extraction

**Figure 1:**
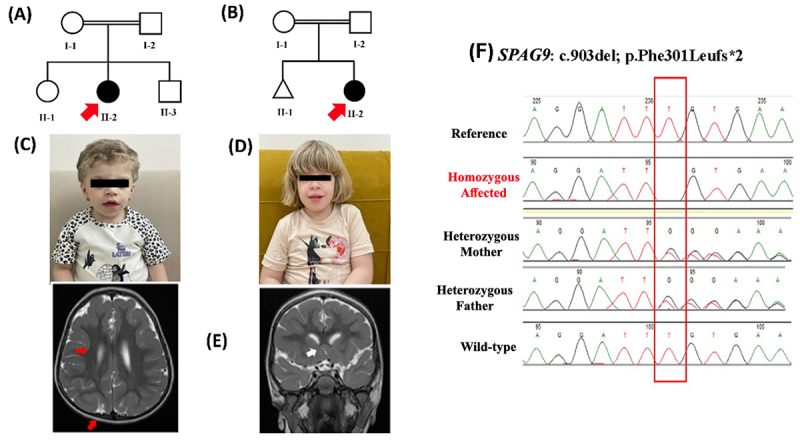
(A, B) Pedigree of Family A and B showing consanguineous union. Both the families had single affected proband. The red arrow indicates the affected individual. (C, D) Images of the affected individuals in both the families A and B. Facial features clearly showing coarse facial features, albinism and golden hairs (E) Brain MRI of patient (II-2) from family A, showing bilateral diffused supratentorial pachygyria, involving the frontal lobes that might be the cause of developmental delay. (F) Sanger sequencing results of the identified *SPGA9*-homozygous frameshift variant (c.903del; p.Phe301Leufs*2) in both the families (Reference (Wild type), Index (homozygous affected), Mother and Father (Heterozygous Carriers).

Two families A and B from Saudi Arabian population with a severe autosomal recessive oculocutaneous albinism were recruited for the current investigation (Figure 1 A , B). Both families were unrelated according to the family history. A detailed medical history was obtained, magnetic resonance imaging (MRI), and biochemical tests were performed (Figure 1 C  -E). Blood samples were obtained from both families (Figure 1 A, B) and processed further for DNA extraction and quantification using conventional techniques (Al Tuwaijri *et al*., 2022).

### Molecular Investigation

Whole genome sequencing (WGS) was performed using standard methods on DNA from both families. The Twist Human Core Exome Plus kit was used to enrich target regions from fragmented genomic DNA by using double-stranded DNA capture baits against approximately 36.5 Mb of the human coding exome (targeting >98% of the coding RefSeq: GRCh37/hg19). The generated library was sequenced on an Illumina platform to achieve at least 20× coverage depth for over 98%. We used an in-house bioinformatics pipeline that included read alignment to the GRCh37/hg19, variant-calling, annotation, and comprehensive variant filtering. The variant calling file (VCF) files were analyzed using Illumina BaseSpace, and different filters were applied based on clinical information and family history (Alhamoudi et al., 2020; Al Hawsawi et al., 2022).

All variants with a minor allele frequency (MAF) of less than 1% in the gnomAD database, as well as disease-causing variants from HGMD®, ClinVar, or CentoMD®, were considered. The search for relevant variants was restricted to coding exons and flanking +/-20 intronic nucleotides of genes with clear gene-phenotype evidence (OMIM®). All possible modes of inheritance patterns were considered; however, based on the pedigree, autosomal recessive was prioritized. ACMG classifies variants into five categories (pathogenic, likely pathogenic, variant of uncertain significant (VUS), likely benign, and benign) (Umair et al., 2020). Using standard screening procedures, we looked for functional variations that could be associated with the patient phenotype. Priority was given to genes that have previously been described in the literature (PUBMED).

### *In silico* analysis

Several techniques were used to determine the discovered variant’s pathogenic potential. To determine if the variant is reported in the general population or not, gnomAD were searched. Using NCBI-HomoloGene, amino acid conservation was determined.

### Sanger sequencing

The observed variant was Sanger sequenced in all accessible members of the family. Sanger sequencing was carried out using standard procedures (Alfadhel *et al*., 2022). Primer pairs were created using an online software application called Primer3. Primer sequence for the Sanger sequencing: [*SPAG9*-903-F1-TTAGCCAAGGCGGATCTAAA, *SPAG9*-903-R1-TCCTGGGCTACCTGTACTTCA] (Figure 1F).

### RNA extraction 

PBMCs were used to isolate total RNA, which was then separated into its organic and aqueous phases using the TRIzol® reagent and chloroform. The RNA from the aqueous phase was transferred to the RNase-free tube after spinning at 4 °C for 15 minutes. After washing with isopropanol, precipitation was done using 75% ethanol. Total RNA was extracted using the RNeasy Plus Mini Kit from Qiagen Inc., purity tests for RNA and quantification were done using conventional methods (Alfadhel *et al*., 2023; Umair *et al*., 2024).

### Quantitative real-time PCR [qRT-PCR]

Total RNA was collected to quantify the expression of *SPAG9* mRNA in comparison to the internal control gene (*GAPDH*). The high-capacity cDNA reverse transcription kit (Applied Biosystems) was used to create cDNA using standard techniques from total RNA (Asiri *et al*., 2022; Al Tuwaijri *et al*., 2023). The *SPAG9* cDNA primer sequences were created using the Primerbank database [*SPAG9*-cDNA-F1: TCTGATGTTAGCCAAGGC, *SPAG9*-cDNA-R1: TTCCTGGGCTACCTGTAC]. Thermo Fisher’s PCR SYBRGreen Master Mix was used in the qPCR reaction, which was run on an Applied Biosystems QuantStudio 6 Flex Real-Time PCR System. The expression Suite software, version 1.1 (Applied Biosystems), was used to analyze the data after each reaction was independently replicated and carried out in triplicate. *GAPDH* was used as the endogenous control, and the PCR cycle settings were according to standard protocols. GraphPad Prism (version 8.1) was used to analyze the quantitative real-time PCR results as the mean standard deviation (SD). For significance evaluation, a t-test with a threshold of p < 0.01 was employed on the samples.

### *SPAG9* sequence retrieval and 3D structure prediction

In this study, *in silico* methodologies such as homology modeling for wild-type and mutant were carried out. The crystal structure of the human C-Jun-amino-terminal kinase-interacting protein 4 (JIP4) was retrieved from the AlphaFold Protein Structure Database (AlphaFold DB), and the Swiss-Model was used to create the structure of the mutated protein. The protein structure from the AlphaFold DB was used as a template to yield the mutated protein structure. The model was then run through the Ramachandran plot server and ERRAT. STRING was used to predict protein-protein interactions (https://www.expasy.org/resources/string).

### Electron microscopic (EM)

Electron microscopic (EM) studies were performed including high resolution imaging of ultra-structural organelles using standard protocols. The specimens were received in glutaraldehyde (to preserve cellular structures at the ultrastructural level). After fixation, the samples are dehydrated with increasing concentrations of alcohol and embedded in resin for sectioning. It was labeled “skin punch biopsy” and consists of a single tiny piece of grayish white soft tissue measuring 0.2 × 0.1 cm in diameter and 0.2 cm in depth. Samples were stained by using uranyl acetate and lead citrate and examined under electron microscope.

## Results

### Clinical Description

*Family A*

The affected individual in family A (II-2) was a 4 years old female, known to have G6PD deficiency, second child of consanguineous parents (Figure 1C). The patient was born following uneventful gestation and delivery, at term. Her APGAR score was 9 and 9 at 1 and 5 minutes respectively, birth weight was 3.4 kg (25^th^-50^th^ percentile), length was 53 cm (90^th^ percentile) and head circumference 34.2 cm (25^th^-50^th^ percentile), and found to have oculocutaneous albinism since birth and she was discharged with her mother in good health. At age of 6 months, she presented with cataract and developmental delay, mainly motor as she has poor head control and cannot sit without support. At one year of age, she underwent cataract surgery and developmentally still cannot sit or crawl and has no speech. She had normal hearing. Her past medical history was consistent of two attacks of pneumonia, one of which was at age 20 days with neonatal intensive care unit (NICU) admission and the other admission was at the age of 2 years. As for her current development (age 4 years), she still cannot walk, she can only sit and crawl. Her soft motor skills are delayed as well; she cannot draw shapes, feed herself, nor help in dressing or undressing or control her sphincters. As for her speech, she can only call her parents, but no other understandable words. Moreover, she cannot follow any commands and does not recognize her siblings well. She is developmentally functioning at age of 9 months.

At her last physical evaluation; the affected individual had weight of 13 kg (3^rd^-5th percentile), length was 91 cm (<3percentile) and head circumference 49 cm (25^th^-50^th^ percentile). General examination revealed features of coarse facial dysmorphism (Ocular hypertelorism with prominent supraorbital rim, low-set ears, broad nose with flaring of nostrils, deeply grooved philtrum with macroglosia), Eye examination showed normal iris pigmentation, full extra-ocular movement, exotropia at distance in left eye and bilateral positive red reflex, no nystagmus., generalized skin hypopigmentation and blond-gray hair, eyelashes and eyebrow. She was also noted to have generalized hypotonia with normal power and reflexes (Figure 1 C). Other system examination were unremarkable. Her echocardiogram showed tiny foramen ovale and mild tricuspid valve regurgitation. Abdomen ultrasound revealed diffuse increased parenchymal liver echogenicity. The skeletal survey was remarkable for mild decrease in bone density and bilateral developmental dysplasia of the hip (DDH) with bilateral coxa valga deformity. Brain Magnetic Resonance Imaging (MRI) was notable for diffuse bilateral supratentorial pachygyria, predominantly involving the frontal lobes (Figure 1 E).

*Family B*

The affected individual in family B (II-2) is 8 years old female born to a consanguineous parent (Figure 1 D). She is a product of full term, delivered by caesarian section (C\S) due to failure to progress. The fetus (II-1) had a spontaneous abortion; however, the reason was not documented. The patient was born following uneventful gestation and delivery, at term. Her APGAR score was 9 and 9 at 1 and 5 minutes respectively, birth weight was 3 kg (25^th^-50^th^ percentile), length was 52 cm (75^th^-90th percentile) and head circumference 34 cm (25^th^-50^th^ percentile), Since birth she was noted to have oculocutaneous albinism, and was admitted to nursery to rule out sepsis with respiratory difficulties given oxygen through nasal cannula and discharged after 10 days with diagnosis of transient tachypnea of newborn. Since discharge, she had hypotonia, feeding difficulties, and frequent vomiting. Also, at 6 months of age, the family noted decreased visual acuity, and her ophthalmological assessment revealed that she had cataract, which was operated on at the age of 10 months. In addition, she has had global developmental delay mainly motor as she has poor head control and cannot sit without support. She sat at two years of age, crawled at 2 year 2 months of age and walked independently at 4 years of age. She also had delayed speech and her hearing was normal. Extensive investigations at the time did not reveal a genetic cause for her presentation including: chromosomal analysis, CGH microarray and whole exome sequencing. Currently she is having global developmental delay, she can ambulate independently on flat surfaces; however, she still needs support to climb up or down the stairs. As for her fine motor (age 8 years), she can only scribble with a pen and cannot draw shapes or letters. She cannot feed herself nor she can maintain her personal hygiene and she is not toilet trained. She needs help in all daily life activities. She can now speak two-words sentences with around 50% understandable words; she can follow only few simple commands. She developmentally functioning at 3 years of age. She also has recurrent otitis media and ear effusion with sleep apnoea; therefore, she underwent adenoidectomy with bilateral myringotomy and right ventilation tube (VT) insertion at 6 years of age. 

**Figure 2:**
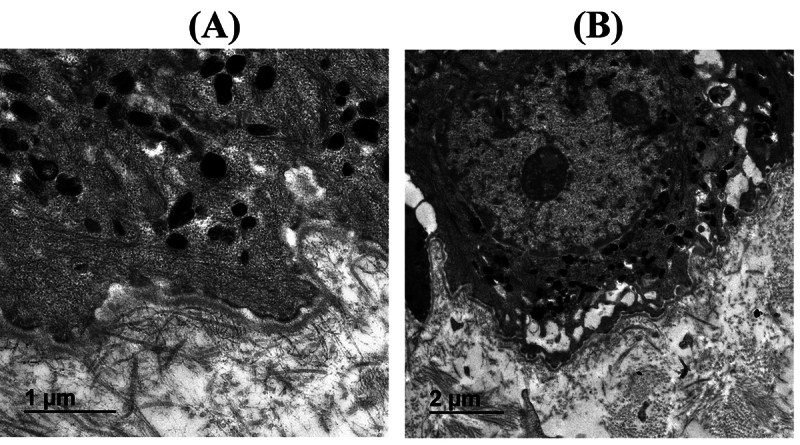
(**A, B**) Electron microscopy for the patient’s skin biopsy (II-2; Family A), suggesting melanocytes with melanosomes of variable stages of development some are lacking melanin pigmentation (pale color).

At her last physical evaluation; she had weight of 30 kg (50^th^-75^th^ percentile), height was 128 cm (25^th^-50^th^ percentile) and head circumference 51 cm (25^th^-50^th^ percentile). She was noted to have features of coarse facial dysmorphism (Ocular hypertelorism, downward slant of palpebral fissure, broad nose with depressed root, deeply grooved philtrum with full lips). Eye examination showed normal iris pigmentation, she is wearing glasses with full extra-ocular movement, squint and bilateral positive red reflex and nystagmus. She has generalized skin hypopigmentation and blond-yellow hair, eyelashes and eyebrow. She was also noted to have generalized hypotonia with normal power and reflexes. The skeletal survey showed right sided developmental dysplasia of the hip (DDH). Hearing assessment was done and was remarkable for bilateral abnormal middle ear function. Her echocardiogram was positive for small patent ductus arteriosus (PDA) and mild tricuspid as well as mitral valves regurgitation. Abdomen ultrasound revealed heterogeneous liver echotexture. Brain MRI and nerve conduction studies were unremarkable. Electron microscopy of the index skin biopsy suggesting melanocyte with melanosomes of variable stages of development some are lacking melanin pigmentation. Electron microscopy of the index skin biopsy suggesting melanocyte with melanosomes of variable stages of development some are lacking melanin pigmentation (pale color; Figure 2 A, B).

### Biochemical investigations

All biochemical investigations were unremarkable including: acylcarnitine profile, plasma amino acids, urine organic acids, creatine kinase (CK) level, total homocysteine, lactic acid, ammonia level, carbohydrate deficient transferrin (CDT) for both the families (A, B).

### Molecular investigation

**Figure 3:**
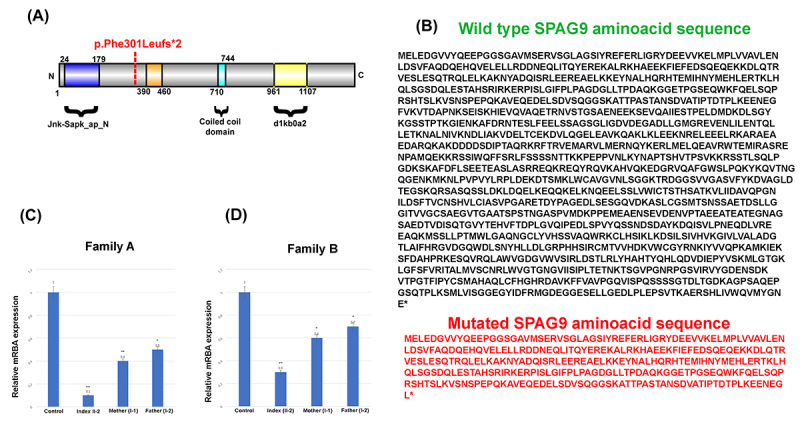
(**A**) Protein domains of SPAG9 showing location of the identified mutation. (**B**) The *SPAG9* consists of Jnk-Sapk_ap_N domain that spans from 24 amino acids (aa) -179 aa, Coiled-coil domain (710aa-744aa) and d1kboa2 (961aa-1107aa). (**C**, **D**) RT-qPCR results of the *SPAG9* in the two families showing reduction of expression in the affected individuals as compared to the parents and control.

The WGS and variants filtration stages were carried for both the families using the previously published standard procedures (Barhoumi et al., 2019). A novel homozygous frameshift variant [c.903del; p.(Phe301Leufs*2)] was identified in both the families in the exon 6 of the *SPAG9* gene located on chromosome 17q21.33 [NM_003971.6]. The variant [c.903del; p.(Phe301Leufs*2)] was discovered after screening and filtering several homozygous and compound heterozygous variants. The *SPAG9* variant [c.903del; p.(Phe301Leufs*2)] creates a shift in the reading frame starting at codon 301 (Figure 3 A, B). The new reading frame ends in a stop codon 1 positions downstream. Sanger sequencing was performed for both families A and B and the variant (c.903del) segregated perfectly with the disease phenotype (Figure 1 F). Moreover, 1500 control genomes were screened for the mutation, and it was discovered that the altered amino acid was conserved in all tested genomes. According to ACMG categorization, the discovered variation is categorized as a variant of uncertain significance (VUS) class 3.

### Quantitative qPCR

The *SPAG9* mRNA expression was examined using RT-qPCR in both families A and B including the probands, both parents, and normal individuals. The RT-qPCR data was analyzed and revealed that the probands (Family A (II-2) and Family B (II-2) having the homozygous variant (c.903del) revealed a significant reduction in the relative mRNA expression of the *SPAG9* as compared to the heterozygous parents and wildtype controls (Figure 3C, D).

### *SPAG9*-3D protein modeling

**Figure 4:**
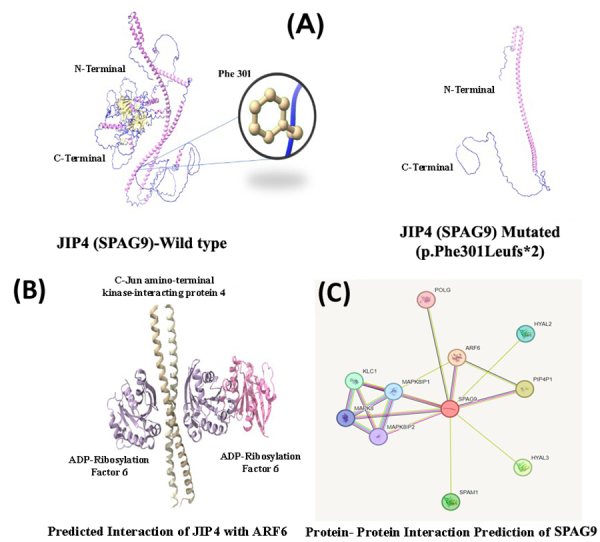
Protein homology and predicted protein-protein interaction of C-Jun-amino-terminal kinase-interacting protein 4. (**A**) 3D protein structure of JIP4 [*SPAG9*] (both wildtype and mutated) are shown. As the mutation is a frameshift that results in a premature stop codon, thus we got a truncated SPAG9 protein. (**B**) Protein-protein interaction of SPAG9 with *ARF6*, *MAPK8*, *MAPK8IP1*, *SPAM1*, *HYAL3*, *HYAL1*, and *PIP4P1* predicted by STRING, thus suggesting important role in different pathways. (**C**) Predicted interaction of JIP4 [*SPAG9*] (wild type) with *ARF6* is significant in regulating cellular dynamics, particularly in processes like membrane trafficking, cytoskeletal reorganization, and cell signaling. As a result of the truncated protein, these interactions will be demolished.

The JIP4 protein has a transmembrane binding site, coiled coil regions, a leucine zipper motif, and a JNK binding motif. The Phenylalanine (F) to Leucine (L) codon substitution brought about by the frameshift mutation that finally led to the protein chain’s termination at amino acid number 302. As a result, it is anticipated that the modified structure will stop at 302 (Figure 4 A). Numerous interactions between these domains and proteins are known to occur. The final revised model was examined by several evaluation programs. According to the Ramachandran plot, the allowed torsion angle zones are occupied by 97% and 99% of the residues in the wild-type and mutant structures, respectively (Figure 4 B).

It is plausible that SPAG9 isoforms might interact with proteins that regulate the trafficking of melanosomes or melanosomal proteins required for appropriate pigmentation since they can interact with proteins like *ARF6*, *MAPK8*, *MAPK8IP1*, *SPAM1*, *HYAL3*, *HYAL1*, *SMCO1* and *PIP4P1* that are involved in certain types of vesicular trafficking (Figure 4 C). Melanins, the colors for skin, hair, and eyes, are created in melanosomes and then carried to the terminals of melanocyte dendrites and exported to nearby keratinocytes through microtubule and actin filaments. The shortened protein caused by the mutation (p.Phe301Leufs*2) is unable to interact with other proteins.

## Discussion

In the present study, we genetically and clinically examined two unrelated families each having single affected individual with oculocutaneous albinism features having fair hair, fair skin, ocular abnormalities, cataract, developmental delay, developmental dysplasia of the hips and coarse facial dysmorphism raising the possibility of a new syndrome. To investigate the molecular cause, the affected individuals were subjected to WGS using standard methods. WGS coupled with Sanger sequencing revealed a novel frameshift variant (c.903del; p.Phe301Leufs*2) in exon 6 of the *SPAG9* gene located on chromosome 17q21.33. *SPAG9* has 29 coding exons, which encode a 1308 amino acid long protein.

Several disorders have been linked to ocular albinism, intellectual disability, and other clinical manifestations. Hermansky-Pudlak syndrome (HPS; OMIM 608233), Chediak-Higashi syndrome (CHS; OMIM 214500), and Nettleship-Falls syndrome (Nettleship syndrome; OMIM 300500) are some common examples. We tested all of the genes associated with these syndromes through WGS and found nothing. Melanin is a pigment that plays a crucial role in the development of the eyes, skin, and hair. There are several syndromes that include oculocutaneous albinism, intellectual disability and other phenotypic presentations. Some of the examples have been presented in Table 1.

**Table 1:** Oculocutaneous albinism with intellectual disability gene defects.

Disorder	Gene	Phenotype MIM number	Clinical description
Hermansky-Pudlak syndrome 1	*HPS1*	203300	Ocular albinism, nystagmus, cardiomyopathy, restrictive lung disease, renal failure, albinism, bleeding diathesis, prolonged bleeding time.
Hermansky-Pudlak syndrome 2	*AP3B1*	608233	Microcephaly, nystagmus, Dental decay, Pulmonary fibrosis, Hepatomegaly, Splenomegaly, intellectual disability.
Hermansky-Pudlak syndrome 6	*HPS6*	614075	Nystagmus, Strabismus, Global developmental delay, Ocular albinism, Frequent nosebleeds, Prolonged bleeding time.
Hermansky-Pudlak syndrome 10	*AP3D1*	617050	Hypotelorism, Nystagmus, Ocular albinism, Microcephaly, Frontal lobe atrophy, Cerebral atrophy, Delayed myelination, Lack of developmental progress, Seizures, refractory, Generalized tonic-clonic seizures, Hepatomegaly, Splenomegaly, Immunodeficiency.
Nettleship-falls type ocular albinism	*GPR143*	300500	Albino pupillary reflex, depigmented fundus, nystagmus, Head nodding.
Chediak-Higashi syndrome (CHS)	*LYST*	214500	Reduced visual acuity, Photophobia, Nystagmus, Strabismus, Hepatomegaly, Jaundice, Mental deficiency, Progressive intellectual decline, Neurodegeneration, Cranial nerve palsies, Seizures, Anemia, Thrombocytopenia.
Holmes-gang syndrome	*ATRX*	309580	Short stature, Microcephaly, Hypertelorism, Exotropia, Ptosis, Optic atrophy, Skeletal defects, Mental retardation, Hypotonia, Hypertonia, Hyperreflexia, Seizures.
Spastic paraplegia, intellectual disability, nystagmus, and obesity	*KIDINS220*	617296	Increased head circumference, Nystagmus, Poor visual acuity, Hypermetropia, Astigmatism, Delayed psychomotor development, Intellectual disability, Delayed speech, Spastic paraplegia, Hyperreflexia
Albinism-microcephaly-digital anomalies syndrome	*N.A*	203340	Microcephaly, oculocutaneous albinism, and digital anomalies
Present study	*SPAG9*	605430	Coarse Facial Features, Albinism, Cataract, Skeletal abnormalities, and Developmental Delay

*SPAG9* has been associated with functional clusters known to be associated with neurodevelopmental disorders (NDDs) including chromatin organization, nervous system development and revealed co-expression/ interaction with other known candidate genes. Thus, suggesting a stronger influence in the etiology of NDDs (Zhang et al., 2021). Similarly, Acosta-Baena *et al*. (2021) present an abstract describing a Colombian family with 9 affected individual’s revealed homozygous variant (p.Tyr914Ter) in the *SPAG9* with features such as severe intellectual disability (ID), speech delayed, gait disturbance, cerebellar syndrome, cataracts, strabismus, seizures and eyelid ptosis. Brain MRI revealed brain atrophy, hippocampal malrotation, thinning of the corpus callosum and white matter hyperintensities (Acosta-Baena *et al*., 2021). However, their patients do not have oculocutaneous albinism. These findings support the data in the present study that mutated *SPAG9* is a potential candidate gene for the novel syndrome. The previously reported patients’ phenotypes clearly overlap. The OCA phenotypes not seen in the patients reported by Acosta-Baena *et al*. (2021), could be related to a change in the mutant domain (p.Tyr914Ter) as compared to ours (p.Phe301Leufs*2) or other regulatory effects caused by the difference in the position of the identified mutation. 

The *SPAG9* gene has been associated with oculocutaneous albinism in homozygous mutant mice models (http://www.informatics.jax.org/allele/MGI:4442906), which also support our current study. The MGI revealed phenotypes such as diluted coat color, absent skin pigmentation, and decreased activated sperm motility in the homozygous knockout mice models (https://www.informatics.jax.org/marker/MGI:1918084). Similarly, *SPAG9* (MGI: 1918084) showed expressing in different mice organs such as nervous system, visual system, and reproductive system. We clearly observe overlapping of the phenotypes as shown in the knockout mice and humans. Our study suggests *SPAG9* gene as a strong candidate for oculocutaneous albinism, developmental delay, cataract and coarse facial features due to loss of function variant (LOF), however further evidences in humans are needed.

Network biology has emerged as a promising approach to identify drug targets in complex genetic disorders, which could be applicable to understanding the molecular interactions involving *SPAG9* in the novel syndrome identified (Naqvi *et al*., 2023). Similarly, the use of molecular and cellular biomarkers has been pivotal in advancing our understanding of developmental toxicology, providing a framework that could be adapted to explore the pathogenic mechanisms involving *SPAG9* (Dey et al., 2023).

In conclusion, to our knowledge, this is the first time to define the phenotypic spectrum of *SPAG9*-associated with coarse facial features, albinism, cataract, skeletal abnormalities, and developmental delay. We provide evidence that biallelic LOF-variants in the *SPAG9* gene might lead to a novel syndrome in humans

## Data Availability

The datasets generated and/or analyzed during the current study are available in the [LOVD; https://databases.lovd.nl/] repository, [Individual #00443992: https://databases.lovd.nl/shared/individuals/00443992].
